# Using ovine eyes as a readily available source for the isolation and 2D or 3D cultivation of uveal melanocytes

**DOI:** 10.1371/journal.pone.0358056

**Published:** 2026-09-21

**Authors:** Chloe B. Rodgers, Nicole Brace, Sharon Hutchison, Antonia L. Pritchard

**Affiliations:** 1 Centre for the Cellular Microenvironment, Advanced Research Centre, University of Glasgow, Glasgow, Scotland, United Kingdom; 2 Genetics and Immunology Department, Division of Biomedical Research, Institute of Health Research and Innovation, University of the Highlands and Islands, Inverness, Scotland, United Kingdom; Wuhu Hospital Affiliated to East China Normal University, CHINA

## Abstract

**Background:**

Uveal melanocytes can be difficult to isolate and culture *in vitro* due to the number of cell types present in the uvea. Methodologies have been described to isolate these cells from humans, macaques and canines. This process has, however, not been described from other more readily available sources of uveal melanocytes, including the modification and establishment of protocols for 2D and 3D growth of isolated uveal melanocytes.

**Objectives:**

To establish a method for isolation and culture of ovine uveal melanocytes.

**Methods:**

Donor globes were dissected, and the uveal tract isolated. Successive enzymatic disaggregation of cells using were used to extract cells. These isolates were confirmed as being uveal melanocytes by the active production of melanin and co-expression of genes specific to melanocytes, in the pigmentation pathway. Uveal melanocytes were cultured in modified F12 media or smooth muscle growth media (SMGM) and assessed for growth rates and methods for establishment of spheroid cultures were examined.

**Results:**

Melanocytes and retinal pigment epithelial (RPE) cells were successfully isolated from uveal explants. Contaminating cell types were not observed and the uveal melanocytes could be cryopreserved, thawed, and cultures successfully re-established. The cells were confirmed as melanocytes. It was found that ovine uveal melanocytes grew better in SMGM than the modified F12 media used for human uveal melanocytes. Methods were established for successfully culturing cells in 3D spheroids.

**Conclusions:**

This extraction technique allows for generation of large populations of ovine uveal melanocytes in a relatively short period of time. In many regions, ovine eyes are a highly available source of primary cells from the eye, allowing for better research into the cellular characteristics of uveal melanocytes. This technique is therefore a useful tool for future studies into uveal melanocyte biology as a model for other less available species.

## Introduction

The uveal tract of the mammalian eye is made up of the adjoining iris, ciliary body and choroid (S1 Fig). The biological processes in uveal melanocytes are less well characterised than in skin melanocytes. Material from human and companion animal eyes are rare due to the infrequency of enucleation. This limits the availability of uveal melanocytes for *in vitro* culture and manipulation, which is further confounded by contamination from other cell types in culture and the slow growth and limited population doublings of these primary cells. As such, the ability to perform relevant experiments on more readily available animal models of uveal melanocytes is an important goal.

Melanocytes within the uveal tract synthesise and store melanin, which plays a critical role in ultraviolet radiation (UVR) and biochemical protection of the non-pigmented cells within the eye. This protection occurs via two separate mechanisms: (a) melanin directly absorbs both visible and UVR light over a range of wavelengths and (b) melanin has antioxidant and free radical scavenging properties [1]. Other cells in the eye produce melanin prenatally: embryonic iris, ciliary body and retinal pigment epithelial cells (IPE, CPE and RPE, respectively); postnatally these cells do not produce melanin [2]. With each cell division after embryonic development, the granules are diluted across daughter cells, resulting in the gradual loss of pigmentation granules in adult primary IPE, CPE and RPE cells [3]. In human uveal melanocyte culture, the melanin content decreases quickly in the early stages of culture, stabilises during active growth and accumulates when cells become senescent [4]; melanin can therefore still be produced by cultured melanocytes. Melanocytes derived from the iris, ciliary body and the choroid have similar melanin content [4].

Uveal melanocytes can be difficult to isolate and culture *in vitro* due to the number of cell types present in the uvea. Following a paper describing a protocol using eyes from macaques [5], a series of papers by Hu *et al*. refined a methodology to isolate and culture uveal melanocytes from human adult donor eyes [4,6,7]. This methodology has since been further described from humans (e.g., [8–12]) and canines [13], however, this process has not been described from other, more readily available, sources of uveal melanocytes.

The aim of this study was therefore to develop a technique for extraction, isolation and culture of ovine uveal melanocytes, derived from an abattoir source.

## Materials and methods

Institutional ethical approval was given for this project (OLETHSH285). The protocol described in this peer-reviewed article is published on protocols.io (https://dx.doi.org/10.17504/protocols.io.j8nlk96x6v5r/v1) and is included for printing purposes as S1 File.

### Globe collection

Sheep eye globes were obtained from the local abattoir on the morning of processing. The eyes were placed on ice for transport and were sectioned within an hour of collection; the total time post-mortem was therefore between 2–3 hours. Before dissection, residual tissue was trimmed and the eyeballs were sprayed with 70% ethanol to prevent fungal infection in cell culture (S2 Fig). Retinal pigment epithelial (RPE) cells were isolated as described in the S1 Materials.

### Isolation of uveal melanocytes

An initial incision was made through the sclera with a scalpel and the globe was cut circumferentially into the anterior and posterior sections using scissors (S2 Fig). The anterior and posterior sections were placed in culture dishes and the vitreous fluid and lens removed (S2 Fig).

### Iris and ciliary body sections

Using tissue dressing forceps, the iris and ciliary body were removed together from the anterior section and placed in a fresh culture dish (S2 Fig). The ciliary body was distinguished from the iris by the difference in colour between the tissues when viewing the anterior side of the removed tissues; the iris was visibly brown in contrast to the black surrounding tissues, the ciliary body. The ciliary body was removed using scissors and placed in a fresh culture dish (S2 Fig).

The iris and ciliary body were washed with magnesium and calcium-free Hank’s Balanced Salt Solution (HBSS; Gibco^TM^) and incubated with 0.25% trypsin solution (Gibco^TM^) at 37°C for 1 hour (iris) and 2 hours (ciliary body).

### Choroidal section

Using dissecting forceps, the retina was removed from the posterior section of the globe, which reveals the RPE layer, including the tapetum lucidum (S2 Fig). To isolate the RPE cells, the posterior section was then filled with 0.125% trypsin-EDTA solution and incubated for 1 hour at 37°C (S2 Fig). The RPE was removed from the posterior cup by agitation, with forceps, until it dispersed into solution (S2 Fig) and prepared for culture (S1 Methods). The choroid was then removed from the sclera and the sclera discarded.

### Isolation of uveal melanocytes from iris, ciliary body and choroid

The iris stroma, ciliary body, and choroid (collectively, the ‘uveal stroma’) underwent successive incubations and cell release, as follows:

A The uveal stroma was incubated for 18 hours at 4°C with 3 mL 0.25% trypsin, then 6 mL FIC media (Fisher Scientific; S1 Table) with 10% Foetal Bovine Serum (FBS; Fisher Scientific) added to inactivate the trypsin. The released cells and media were collected, centrifuged at 300g for 5 minutes, then resuspended in 6 mL complete medium (S1 Table) and seeded into three wells of a 6-well plate.
B The uveal stroma was washed with HBSS and incubated with 5 mL collagenase (1.27 mg/mL; Gibco^TM^) in F12 medium with 10% FBS at 37°C for 1 hour. The released cells were collected and pelleted (300g for 5 minutes) and resuspended in 6 mL complete medium (S1 Table) into three wells of a 6-well plate.
C Another collagenase incubation was performed, as described in step B, to maximise the yield; the cells were seeded separately into three wells of a 6-well plate to minimise contamination potential.

In all three steps, geneticin was added to the culture media (final concentration 100 µg/mL) for 7 days to inhibit fibroblasts; thereafter, cells were cultured in medium without geneticin (S1 and S2 Tables). Media was changed approximately twice a week and cells were cryopreserved in media containing 10% DMSO.

### Media investigated for optimal culturing of uveal melanocytes

The media and supplements tested or experimentally used to culture primary uveal melanocytes are summarised in S1 Table. Early studies showed that the addition of 12-O-Tetradecanoylphorbol-13-acetate (TPA) (also known as PMA; phorbol 12-myristate 13-acetate) and cholera toxin to culture medium aided growth of skin melanocytes [14]. The effect of PMA and cholera toxin on uveal melanocytes showed that cell numbers increased, whilst the growth of contaminating cells decreased [5]. Human uveal melanocytes are often grown in FIC medium (S1 Table), another outcome of the Hu et al 1993 protocol, which tested various combinations of supplements added to F12 medium [6,7,10,15–18].

Here, in addition to FIC medium (S2 Table), we tested complete smooth muscle growth medium (SMGM; S2 Table), which was selected as it consistently improved growth rates across commercially available human uveal melanoma cell lines in the Cancer Cell Line Factory (CCLF)/DepMap project [19]. Epidermal growth factor (EGF) and insulin have previously been added to culture media for the growth of skin melanocytes [20] and EGF was included in the CCLF media optimisations [19]. We therefore investigated the addition of 10 µg/mL human insulin (I2643, Sigma) and 10 ng/mL EGF Recombinant Human Protein (Gibco™; Fisher) to the SMGM media.

### Measurement of melanin content

Total melanin content per cell was quantified spectrophotometrically using synthetic melanin as a standard as previously described [21]. Uveal melanocytes were seeded into 12-well plates. Three, five and twelve days after seeding, cells were trypsinised and counted using a haemocytometer. The cells were pelleted (500g for 5 minutes) and lysed with 1% Triton-X100 in PBS for 30 minutes at 4°C, then pelleted (500g at 4°C for 30 minutes). The soluble fraction was removed, and protein content measured using the Micro Total Protein Kit (Sigma, UK). The insoluble fraction containing the melanin was solubilised by adding 400 µL 1N NaOH and incubated for 1 hour at 85°C. This fraction was immediately read at λ = 405nm (Varioskan™ LUX multimode microplate reader (ThermoFisher, UK) and melanin content was measured against a standard curve for synthetic melanin (M8631, Sigma, UK) at a range of 0.012–100 µg/mL.

The active expression of the genes encoding tyrosinase (*TYR*), premelanosome protein (*PMEL*) and melan-A (*MLANA*) were assessed by the PCR of mRNA (details in S1 Methods).

### Culture of 3D spheroids

The creation of 3D spheroids of uveal melanocytes was investigated using various methods including the hanging drop method, agar-coated plates, ultra-low attachment plates and round-bottom plates (details in S1 Methods).

## Results

### Isolation and culture of ovine uveal melanocytes

Uveal melanocytes from the uveal tract (iris, ciliary body and choroid) were successfully isolated, based on the protocol described by Hu *et al*. described in humans [4,6,7]. Melanocyte isolation and growth in the FIC complete medium [6] (S2 Table) was tested first; after approximately three days the uveal melanocytes obtained from sequential trypsin and collagenase treatments began to adhere to the plate (S3 Fig). Cultures became uniformly of a single cell type and grew to ~80–90% confluent after approximately 20 days (S3 Fig) and were visibly pigmented. Given the success of isolation and growth of cells of a single type from each of the sequential trypsin/collagenase treatments, these were combined to provide ‘ovine uveal melanocytes’ for all future experiments. RPE cells were also successfully isolated; the morphology of the uveal melanocytes and the RPE cells were discernibly different (S3 Fig).

### Optimal culture conditions for the growth of uveal melanocytes derived from sheep

**Fig 1:**
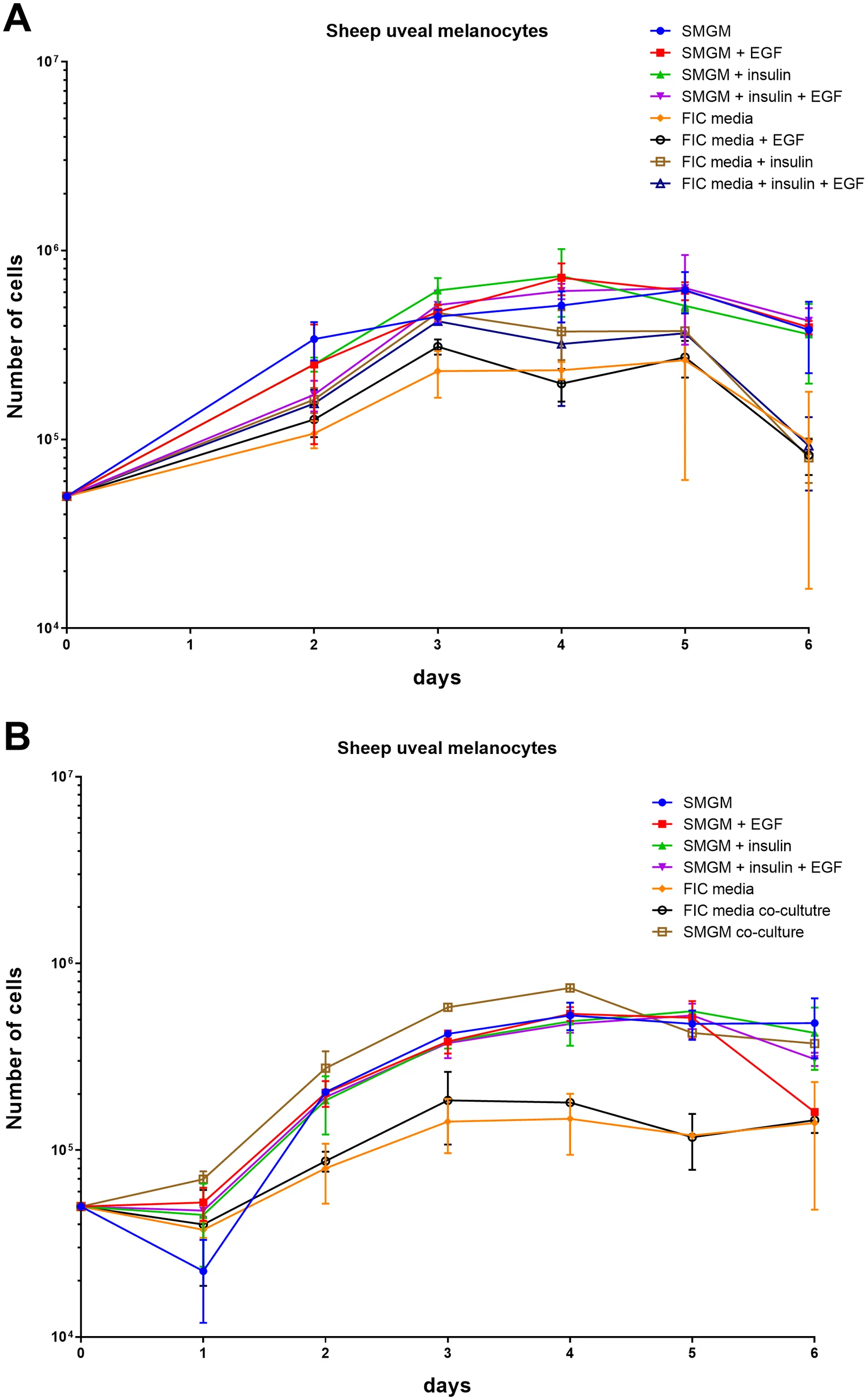
Growth of sheep uveal melanocytes in complete FIC or SMGM media plus insulin and/or EGF. A and B show results from uveal melanocytes derived from the eyes of different sheep. Each panel shows the growth of uveal melanocytes alone in different media (FIC or SMGM), or with the specified additives, and error bars indicate standard deviation of technical replicates (n = 3).

The growth of uveal melanocytes isolated from biological replicates (different eyes, obtained on different days; Figs 1A and 1B) in complete FIC medium (S2 Table) was compared to those grown in complete SMGM (S2 Table). Increased growth of ovine uveal melanocytes was uniformly found in complete SMGM compared complete FIC medium and the growth in FIC medium was more varied between technical replicates (Fig 1). No notable change in proliferation occurred when EGF and insulin additives were added, either separately or together, to complete SMGM media (Figs 1A and 1B).

### Confirmation of uveal melanocyte isolation

#### Melanin production.

Uveal melanocytes actively produce melanin pigmentation [2]; therefore the melanin content of the isolated sheep uveal melanocyte was measured to confirm the specific isolation of these cell types.

The melanin content of cells grown in complete FIC and SMGM media, and SMGM media with insulin and EGF added was measured against a standard curve for synthetic melanin. After correction for total cell protein content, each of these cultures had a similar melanin concentration between all conditions, ranging from 4.66–5.49 µg/ml (Table 1).

**Table 1:** Melanin contents of isolated uveal melanocytes.

Uveal melanocytes growth conditions	Cell counts	Melanin ng/mL	Melanin ng/cell	Protein content (ng/mL)	Ratio of melanin (ng/ml) / protein concentration (ng/mL)
**SMGM**	4.1x10^5^	4659.2	0.0114	775007.9	0.0060
**SMGM + EGF**	4.05x10^5^	5237.2	0.0129	690484.7	0.0076
**SMGM + insulin**	3.7x10^5^	5170.0	0.0140	801251.2	0.0065
**SMGM + EGF + insulin**	3.85x10^5^	4941.5	0.0128	781777.6	0.0063
**FIC media**	1.6x10^5^	5488.8	0.0343	819889.5	0.0067

The cell counts, melanin content, protein content, and the ratio of melanin to protein content in uveal melanocytes grown under different conditions.

To monitor the active production of melanin in uveal melanocytes, the melanin content was analysed at three different timepoints after seeding: day 0, 5, 8 and 12. The ratio of melanin content to total protein concentration (ng/mL) increased modestly over time, demonstrating active melanin production (S4 Fig).

#### Markers of uveal melanocytes.

After confirmation of these cell types via melanin content and active melanin production, we investigated whether this could be established using another common method: staining for the key melanocyte protein markers. No suitable anti-sheep antibody products were found, so anti-human antibodies were investigated due to the relatively high cross-species sequence homology. The anti-human S100B and anti-human SOX-10 antibodies did not stain ovine uveal melanocytes but did stain positive controls (human cutaneous melanoma cell line A2058); this was therefore not an appropriate method for cell type confirmation. Finally, we investigated another method frequently used to identify melanocytes. Ovine specific primers were designed to assess the expression of genes (*TYR*, *PMEL*, *MLANA*) involved in the active production of melanin, a function exclusive to melanocytes, with mRNA co-expression specific to these cells. Each of these genes was robustly expressed by the cells (S5 Fig), providing further evidence that the isolated cells were ovine uveal melanocytes.

### 3D culture ovine uveal melanocytes

The ability to culture primary cells in 3D configuration would significantly improve the utility of ovine melanocytes for wider purposes; we investigated several methods to achieve this.

#### Hanging drop.

**Fig 2:**
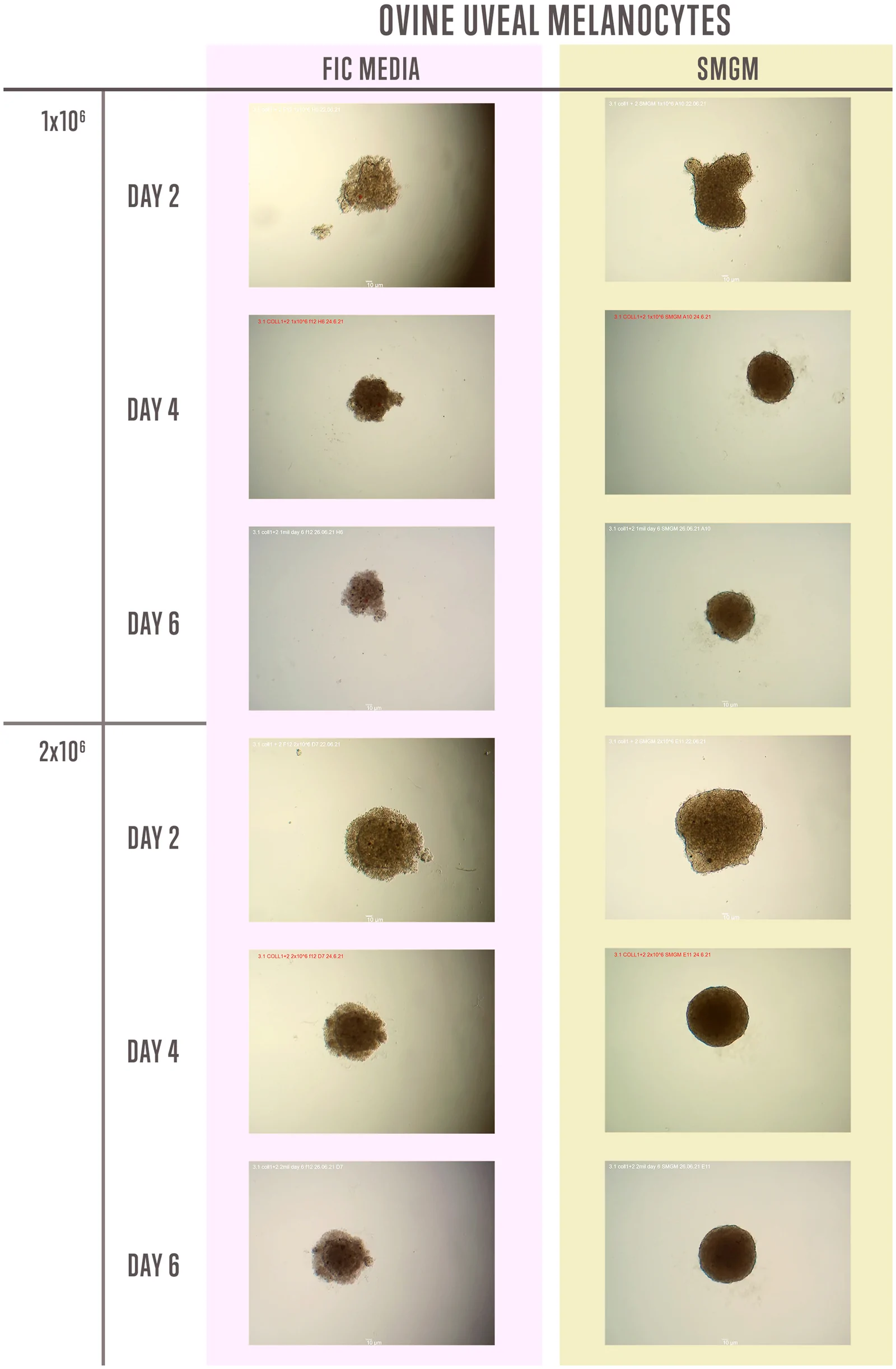
Ovine uveal melanocyte spheroids created using the hanging drop method. Comparison of ovine uveal melanocyte spheroids grown in FIC media or SMGM over 6 days at 1 or 2x10^6^ cells/mL. Representative photographs of ovine uveal melanocytes spheroids created using the hanging drop method and transferred to agar-coated plates (n = 3 replicates were performed). Photographs were taken at 4x magnification.

Uveal melanocytes left in hanging drop for 48 hours developed and transferred more uniformly than those left 24 hours and while both 1x10^6^ and 2x10^6^ cells/mL formed spheroids, the latter were larger and less likely to break apart upon transferring from hanging drop to an agar-coated plate (Fig 2). Spheroids grown in SMGM had a smooth and well-defined round edge compared to those grown in FIC media, which had irregular edges by day 4 (Fig 2).

#### Addition of methyl cellulose to culture media for uveal melanocyte spheroid creation by the hanging drop method.

**Fig 3:**
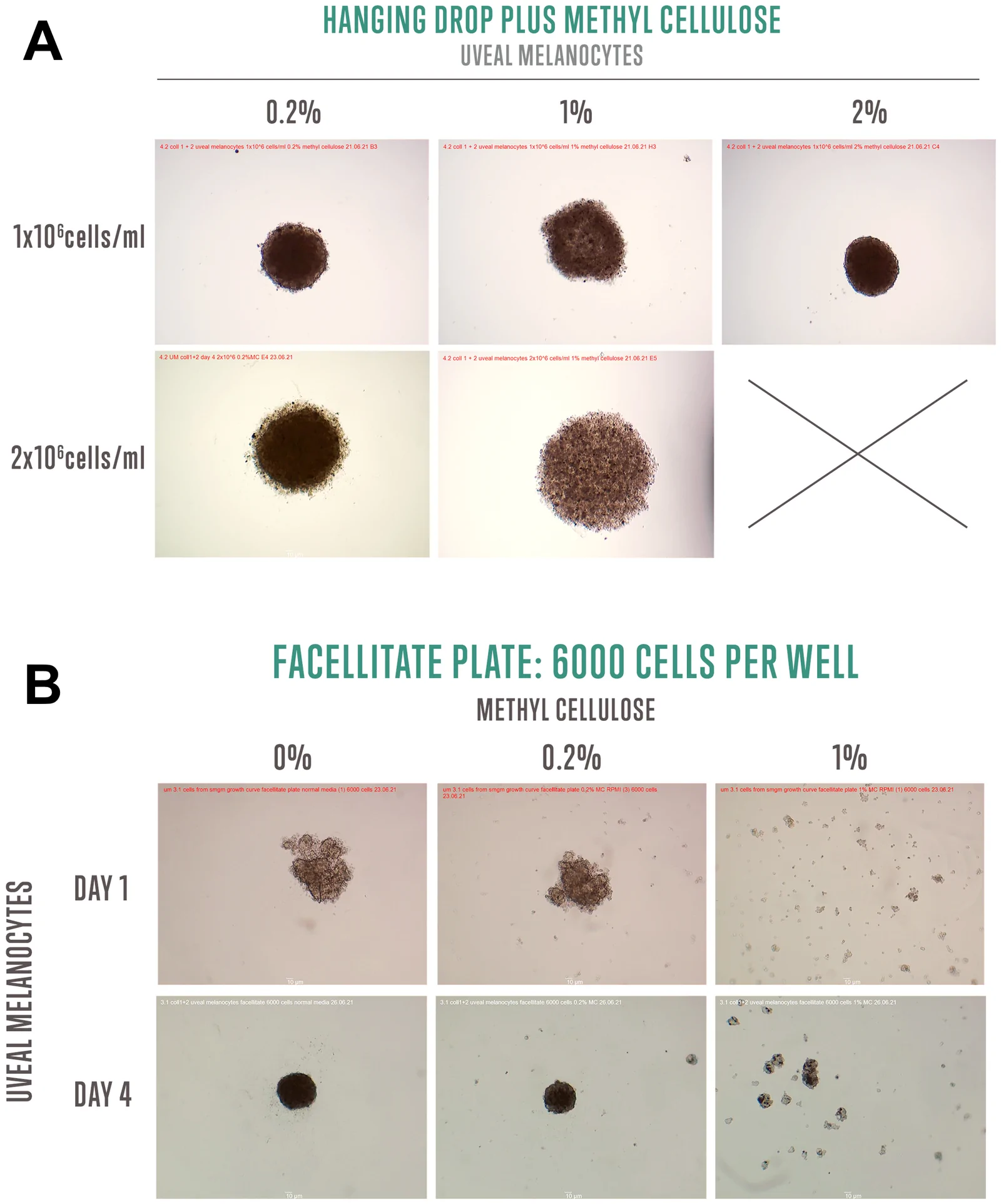
Ovine uveal melanocyte spheroids formed using the hanging drop method with the addition of methyl cellulose to the media. A: Cells were seeded at 1x10^6^ cells/mL or 2x10^6^ cells/mL in complete FIC medium with 0.2, 1 and 2% methyl cellulose. The cross indicates conditions where no cell aggregates could be transferred and therefore no photographs were taken. B: Using faCellitate BIOFLOAT^TM^ round-bottom plates to form spheroids with uveal melanocytes at 6000 cells per well. Photographs were taken at 4x magnification. Photographs were taken 48 hours after cell aggregates were transferred for all photographs.

The addition of 0.2, 1 and 2% methyl cellulose [22–24] to complete FIC media upon transfer from 48 hours in the hanging drop was then tested. 2% methyl cellulose made the media highly viscous and the spheroid often became stuck inside the solidified drop; however, occasionally this method was successful (Fig 3A). Spheroid formation was, however, consistently successful when using 0.2% and 1% methyl cellulose in complete FIC media (Fig 3A) and created more smooth spheroids which were easier to transfer than FIC media alone (Fig 3A).

#### Creation of spheroids using round-bottom plates.

Ovine uveal melanocyte in complete FIC media alone and 0.2% methyl cellulose began to form aggregates after 1 day culture, in faCellitate BIOFLOAT^TM^ round-bottom plates, which then became small, compact spheroids by day 4 (Fig 3B). Uveal melanocytes did not form spheroids in 1% methyl cellulose, but cells remained single cells in suspension suggesting the media was too thick to allow cells to aggregate (Fig 3B).

## Discussion

Ovine eyes were used to obtain uveal melanocytes, as a readily available (abattoir) source of these rare primary cells in many countries. The method described here was based on the work of Hu *et al*. on human eyes [6]. Non-human studies have previously described canine [13] and macaque [5] uveal melanocyte isolation, but this is the first report to describe a methodology for culturing a more readily available source of these cells, including different media formulations and investigations how to best grow these cells as 3D spheroids.

The morphologies between the uveal melanocyte, RPE cell and ‘outgrowth’ cell cultures were dissimilar, indicating different cell types had been isolated. The presence of uveal melanocytes were confirmed by demonstration of active transcription of the key genes encoding proteins involved in pigmentation production, as well as the active melanin production by these cells. The melanin content and production of the isolated ovine uveal melanocytes was similar to values previously reported in human uveal melanocytes during the active growth phase [4]. Similarly, the purity of the cultured cells and duration of the protocol were comparable to the previously described studies on human eyes. While anti-sheep antibodies for the selected melanocyte markers were not available, there are many anti-sheep primary antibodies available, which could assist in better understand the biology of these cells.

Complete SMGM media increased the growth of sheep uveal melanocytes compared to the complete FIC media (which is used to culture human uveal melanocytes) and resulted in more rapid and consistent spheroid formation. This was the media suggested from the CCLF project as the media of choice for culture of human uveal melanomas. SMGM is therefore recommended for the culture of primary ovine uveal melanoma cells and suggests this media should be investigated for other sources of uveal melanocytes also. We focused on manipulation of conditions for spheroid creation using complete FIC media, given the consistency of compact, smooth spheroid formation using complete SMGM media. Each method had its own advantages and disadvantages, based on practicality and consistency. The hanging drop method is inexpensive and does not require specialised plates or equipment, however, requires the transfer of the spheroid from the hanging drop to an agar coated plate within 48 hours and spheroids that are less compact with poor cell-cell adhesion broke apart upon transfer. Spheroids were also created using two types of specialised culture plates: ultra-low attachment plates and faCellitate plates. These plates have the advantage of avoiding spheroid transfer, however, using them for large scale *in vitro* experiments would become expensive. The addition of methyl cellulose to the media improved spheroid compactness, however, in combination with the faCellitate plate resulted in smaller, more compact spheroids, compared to other methods.

Our protocol describing the isolation of uveal melanocytes from ovine eyes provides a platform that will enable researchers to study diseases linked to uveal melanocytes such as uveal melanoma. Moreover, the protocol can be adapted to investigate the interaction of uveal melanocytes with other adjacent cell types, for example, RPE cells. The method can be developed further to include scaffold-based 3D culture, for example, culturing the spheroids in hydrogels. 3D models are invaluable tools to investigate spheroid biomechanics to gain critical insights into the role of biophysics in pathological phenomena. Knowledge of the influence and interplay of surrounding cells and the microenvironment, which allows the growth of metastatic disease and resistance to treatment, may reveal novel targets for therapy or mechanisms to overcome resistance. It is critical to develop improved preclinical cell culture models to allow for more translational experimental analysis of uveal melanoma, particularly because there is a lack of treatment options for metastatic disease. There is potential for the success of immunotherapies, as seen in cutaneous melanoma, to also be beneficial in the treatment of uveal melanoma. However, in order to utilise immunotherapy, the uveal melanoma cells would need to be consistently grown in vitro to allow for high-throughput drug screening, highlighting the importance of our work.

## Conclusion

A method for the isolation of ovine melanocytes of uveal origin, followed by the 2D and 3D culture were successfully developed. This protocol exploits the use of a renewable source of uveal melanocyte primary cells to optimise isolation and growth conditions for these cells, which resulted in the consistent cultures. This protocol may be applied or modified to allow other such readily available sources of uveal melanocytes (for example, such as bovine and porcine eyes) if sheep eyes are less readily available in a given region, to also be used by laboratories with an interest in uveal melanocyte biology.

## Supporting information

S1 Fig Anatomical structure of the eye with an indication of incision to make anterior and posterior sections. (PDF)

S2 Fig The various sections of the dissected eye. Front (A) and back (B) of a sheep eye before dissection. (C)The anterior and posterior sections and removed lens and vitreous fluid from the eye. (D) The remainder of the anterior section once the iris and ciliary body have been removed. (E) The iris anterior side up. (F) The iris posterior side up. This still has the ciliary body attached. (G) The ciliary body was removed and put into a separate petri dish. (H) The posterior section of the eyeball after the retina has been removed. (I) The posterior cup filled with trypsin. (J) The iris was incubated with 0.25% trypsin for 1 hour and the iris stroma was separated (K) The iris melanocyte cells captured in the trypsin, ready for the next step of the protocol.
(PDF)

S3 Fig Cell growth from sequential trypsin and collagenase treatment of ovine eyes. (A) Photographs show the cells obtained from each subsequent treatment: trypsin, collagenase 1 and collagenase 2 at 5, 10, 15 and 20 days post-isolation. Uveal melanocyte cells become the predominant cells and proliferate over this time. Photographs were taken at 4x magnification. (B) Images of the uveal melanocytes and RPE cells finally isolated using the total protocol.
(PDF)

S4 Fig Melanin production over 12 days. The amount of melanin present (ng/ml) per protein content of the cell (ng/ml) were calculated on days 5, 8 and 12 of culture to investigate melanin production by two independent isolations of uveal melanocytes.
(PDF)

S5 Fig Detection of active expression of genes involved in the pigmentation production pathway in isolated ovine melanocytes. The co-expression of genes PMEL, TYR and MLANA are exclusively to melanocytes. (A) The triplicate and median Ct values for each gene, for two separate melanocyte isolations from different source eyeballs. (B) the amplification plots for Sample 1 (red) and Sample 2 (mustard). These data demonstrated robust expression of all three genes by both samples assessed.
(PDF)

S1 Table Media used in published studies for the culture of uveal melanocytes from primary tissue. (DOCX)

S2 Table Components of FIC media and smooth muscle growth media (SMGM) tested for growth of ovine melanocytes. (DOCX)

S1 Methods Supplementary materials and methods. (DOCX)

S1 File Step-by-step protocol, also available on protocols.io. (PDF)
