# Supplementary material for: Using ovine eyes as a readily available source for the isolation and 2D or 3D cultivation of uveal melanocytes
Source: PLoS One. 2026 Sep 21;21(9):e0358056. doi: 10.1371/journal.pone.0358056 (PMC13592627; doi:10.1371/journal.pone.0358056)

Supplementary Figure 1: Anatomical structure of the eye with an indication of incision to make anterior and posterior sections.

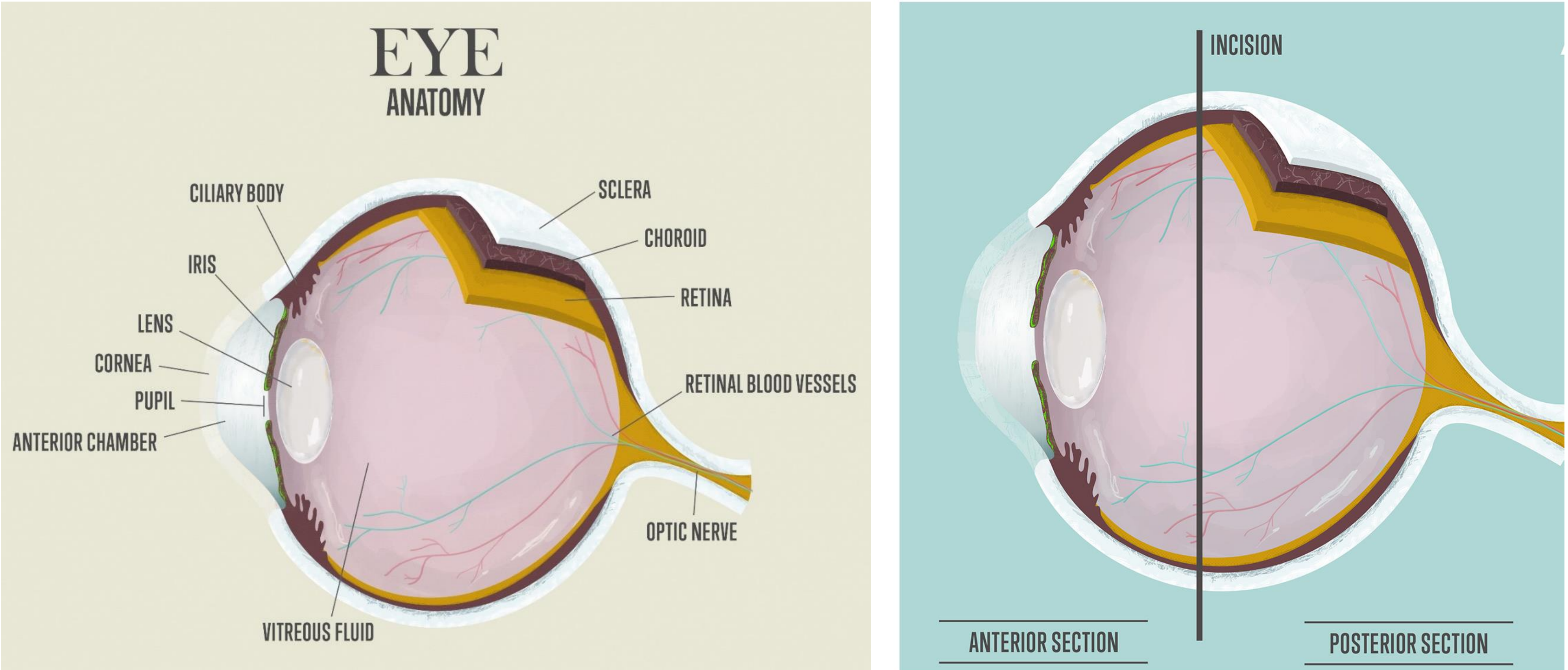

Supplement: Supplementary file 1 [file pone.0358056.s001.pdf]
