# Supplementary material for: Using ovine eyes as a readily available source for the isolation and 2D or 3D cultivation of uveal melanocytes
Source: PLoS One. 2026 Sep 21;21(9):e0358056. doi: 10.1371/journal.pone.0358056 (PMC13592627; doi:10.1371/journal.pone.0358056)
Supplement: Supplementary file 2 [file pone.0358056.s002.pdf]

Supplementary Figure 2: The various sections of the dissected eye.

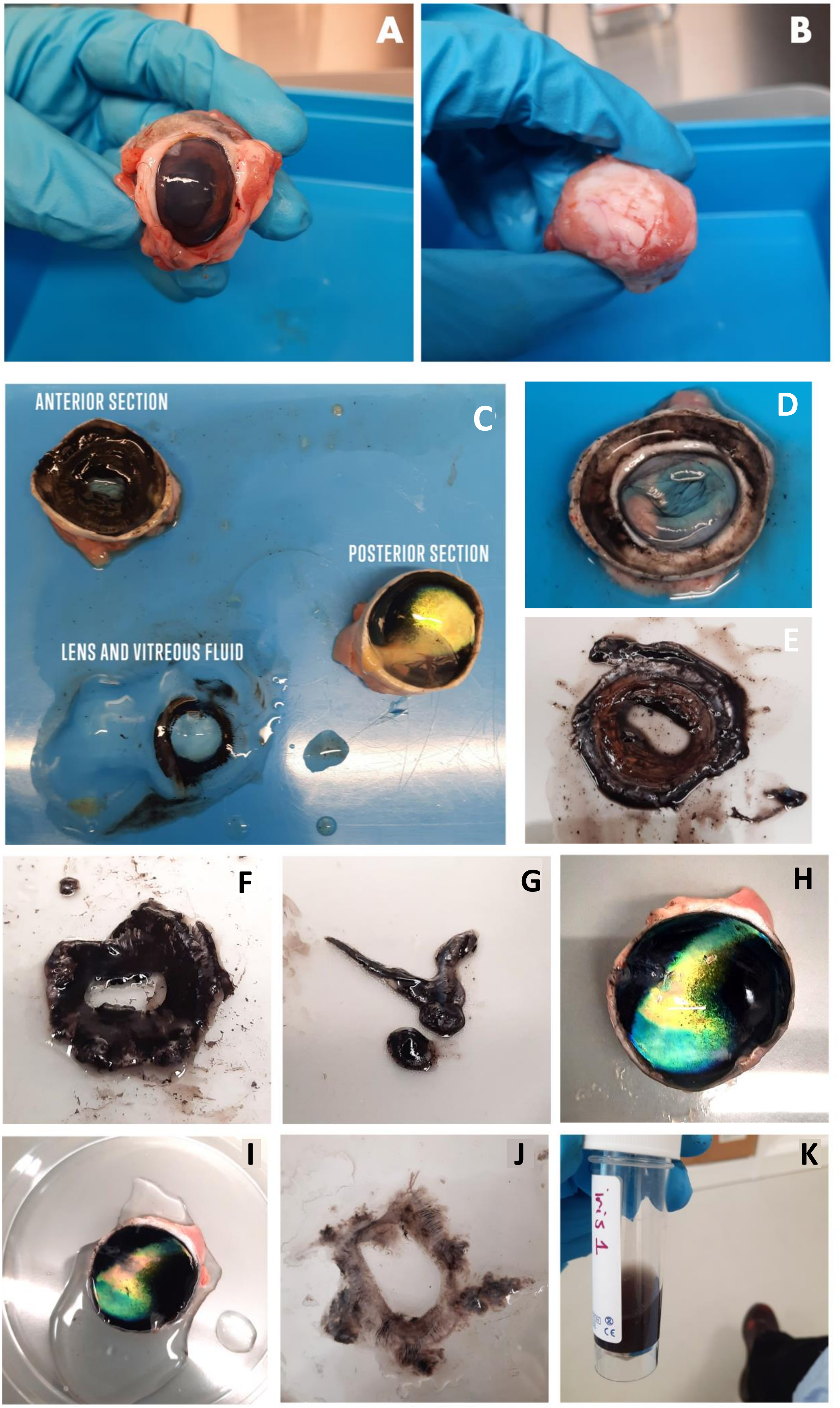

Front (A) and back (B) of a sheep eye before dissection. (C)The anterior and posterior sections and removed lens and vitreous fluid from the eye. (D) The remainder of the anterior section once the iris and ciliary body have been removed. (E) The iris anterior side up. (F) The iris posterior side up. This still has the ciliary body attached. (G) The ciliary body was removed and put into a separate petri dish. (H) The posterior section of the eyeball after the retina has been removed. (I) The posterior cup filled with trypsin. (J) The iris was incubated with 0.25% trypsin for 1 hour and the iris stroma was separated (K) The iris melanocyte cells captured in the trypsin, ready for the next step of the protocol.
