# Supplementary material for: Using ovine eyes as a readily available source for the isolation and 2D or 3D cultivation of uveal melanocytes
Source: PLoS One. 2026 Sep 21;21(9):e0358056. doi: 10.1371/journal.pone.0358056 (PMC13592627; doi:10.1371/journal.pone.0358056)
Supplement: Supplementary file 3 [file pone.0358056.s003.pdf]

Supplementary Figure 3: Cell growth from sequential trypsin and collagenase treatment of ovine eyes.

A. Photographs show the cells obtained from each subsequent treatment: trypsin, collagenase 1 and collagenase 2 at 5, 10, 15 and 20 days post-isolation. Uveal melanocyte cells become the predominant cells and proliferate over this time. Photographs were taken at 4x magnification.
B. Images of the uveal melanocytes and RPE cells finally isolated using the total protocol.

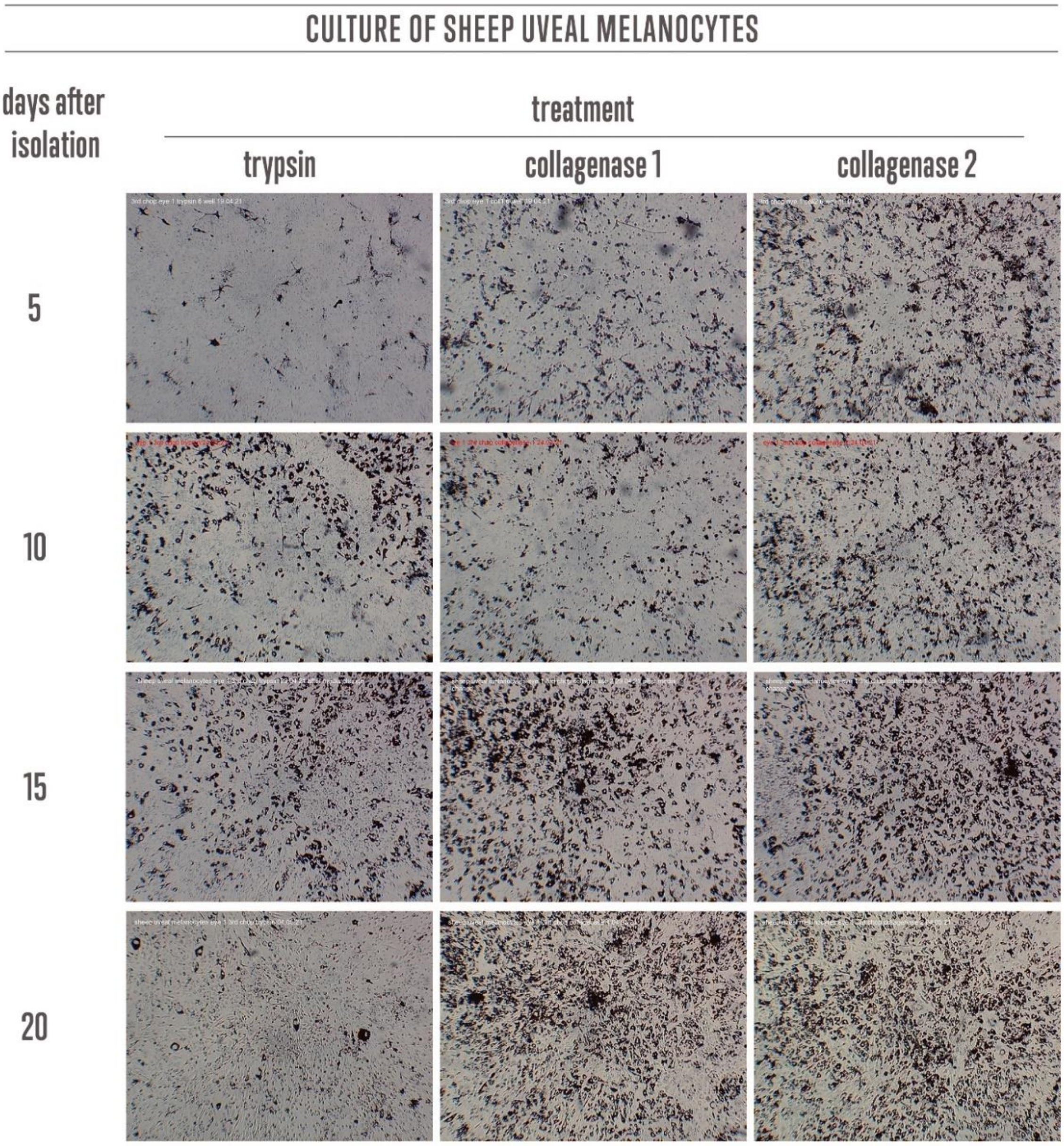

Final isolated cells after one month (30 days) of culture

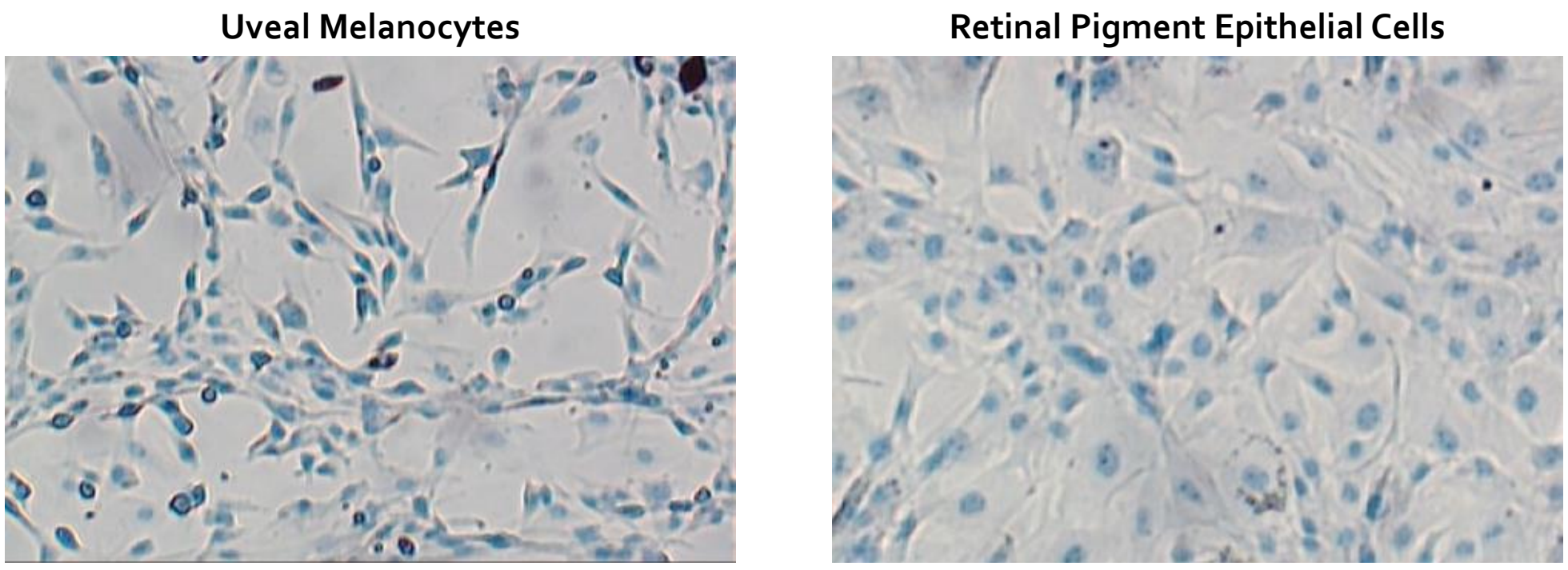

Cells were stained with haematoxylin to visualise the nucleus. Morphological differences are apparent between the uveal melanocytes and the retinal pigment epithelial cells *in vitro* culture.
