# Supplementary material for: Using ovine eyes as a readily available source for the isolation and 2D or 3D cultivation of uveal melanocytes
Source: PLoS One. 2026 Sep 21;21(9):e0358056. doi: 10.1371/journal.pone.0358056 (PMC13592627; doi:10.1371/journal.pone.0358056)

Supplementary Figure 4: Melanin production over 12 days

The amount of melanin present (ng/ml) per protein content of the cell (ng/ml) were calculated on days 5, 8 and 12 of culture to investigate melanin production by two independent isolations of uveal melanocytes.

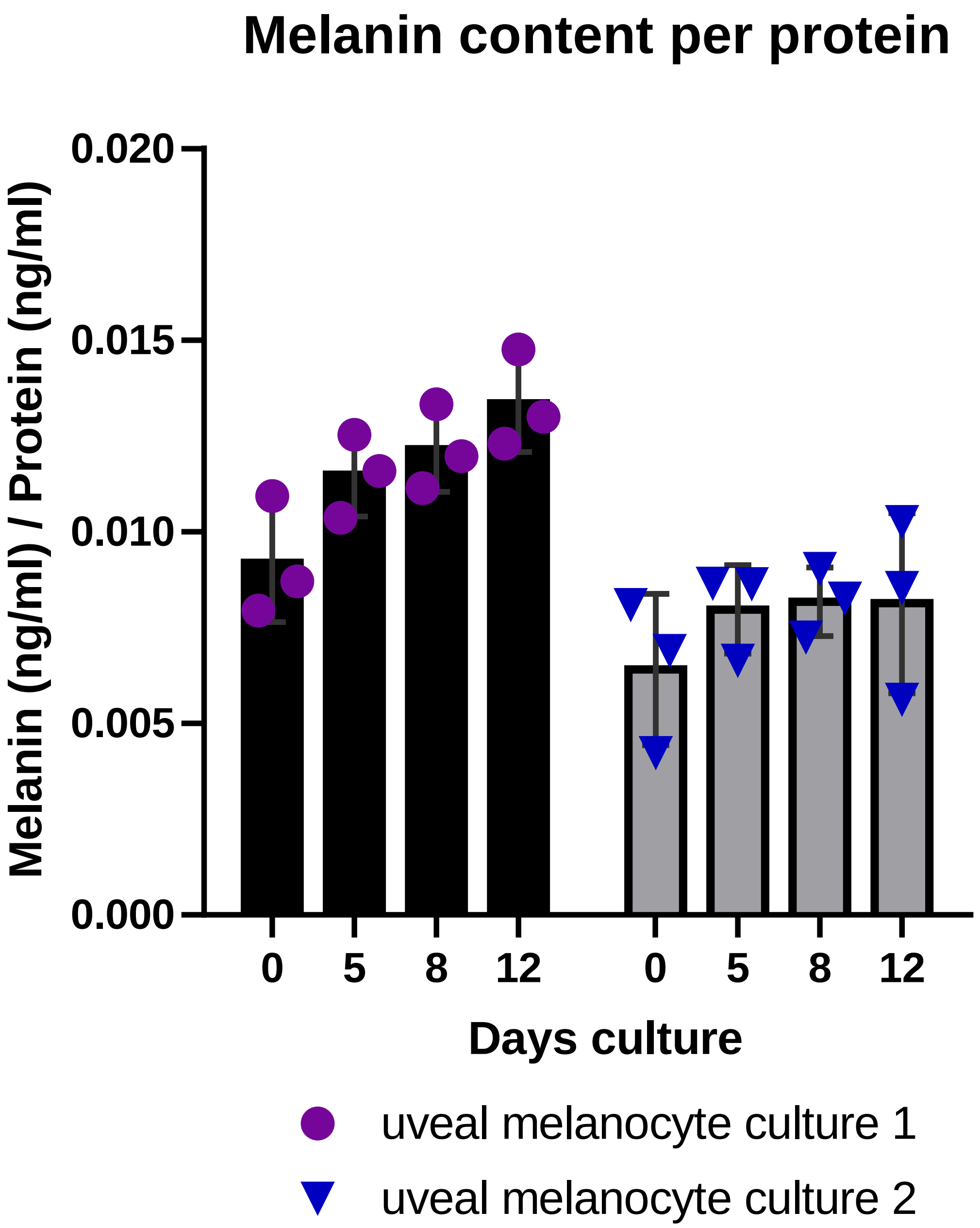

Supplement: Supplementary file 4 [file pone.0358056.s004.pdf]
