# Supplementary material for: Using ovine eyes as a readily available source for the isolation and 2D or 3D cultivation of uveal melanocytes
Source: PLoS One. 2026 Sep 21;21(9):e0358056. doi: 10.1371/journal.pone.0358056 (PMC13592627; doi:10.1371/journal.pone.0358056)

Supplementary Figure 5: Detection of active expression of genes involved in the pigmentation production pathway in isolated ovine melanocytes.

The co-expression of genes *PMEL*, *TYR* and *MLANA* are exclusively to melanocytes. (A) The triplicate and median Ct values for each gene, for two separate melanocyte isolations from different source eyeballs. (B) the amplification plots for Sample 1 (red) and Sample 2 (mustard).

These data demonstrated robust expression of all three genes by both samples assessed.

A

	PMEL			Median	TYR			Median	MLANA			Median
Sample 1	25.98	24.79	24.60	24.79	24.01	24.28	24.24	24.24	21.72	21.96	21.77	21.77
Sample 2	26.9	25.77	26.49	26.49	24.65	24.87	24.60	24.65	24.83	24.40	24.50	24.50

B

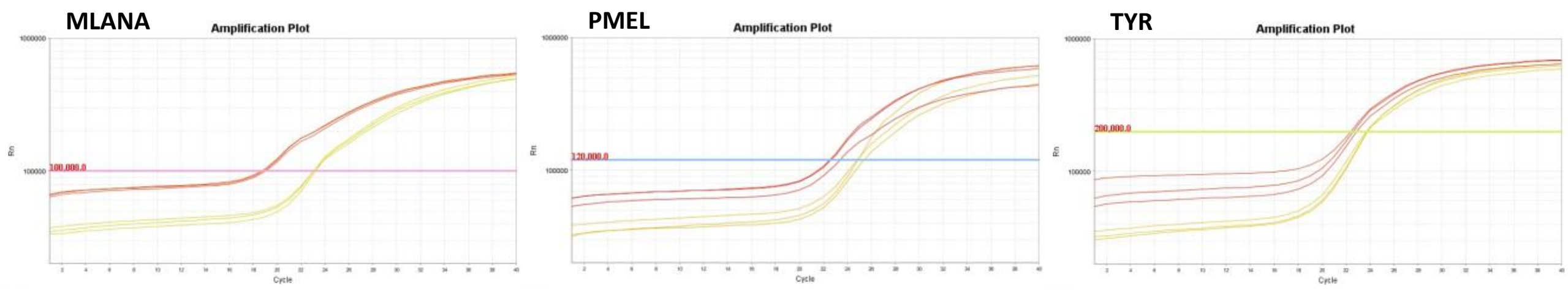

Supplement: Supplementary file 5 [file pone.0358056.s005.pdf]
