# Supplementary material for: Using ovine eyes as a readily available source for the isolation and 2D or 3D cultivation of uveal melanocytes
Source: PLoS One. 2026 Sep 21;21(9):e0358056. doi: 10.1371/journal.pone.0358056 (PMC13592627; doi:10.1371/journal.pone.0358056)
Supplement: Supplementary file 6 [file pone.0358056.s009.pdf]

Aug 18, 2026

Isolation of uveal melanocytes from ovine eyes

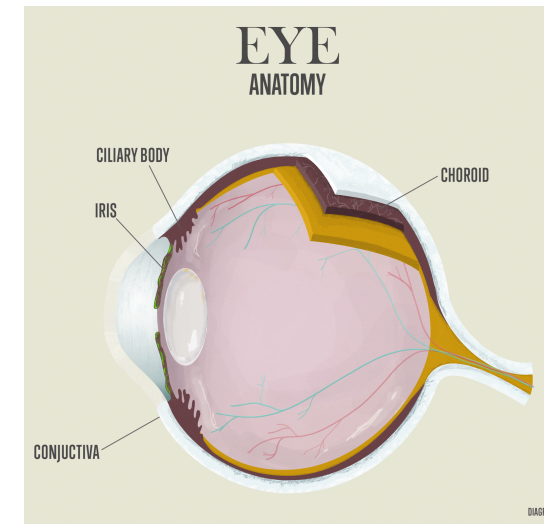

Manage mention

DOI
https://dx.doi.org/10.17504/protocols.io.j8nlk96×6v5r/v1

Chloe Rodgers[1], Nicole Brace[2], Sharon Hutchison[2], Antonia Pritchard[2]

[1]University of Glasgow; [2]University of the Highlands & Islands

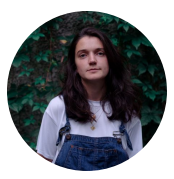

Chloe Rodgers
University of Glasgow

OPEN ACCESS

DOI: https://dx.doi.org/10.17504/protocols.io.j8nlk96x6v5r/v1

Protocol Citation:
Chloe Rodgers, Nicole Brace, Antonia Pritchard 2026. Isolation of uveal melanocytes from ovine eyes. **protocols.io** https://dx.doi.org/10.17504/protocols.io.j8nlk96x6v5r/v1

Manuscript citation:

License: This is an open access protocol distributed under the terms of the **Creative Commons Attribution License**, which permits unrestricted use, distribution, and reproduction in any medium, provided the original author and source are credited

Protocol status: Working
We use this protocol and it's working

Created: January 16, 2025

Last Modified: August 18, 2026

Protocol Integer ID: 118597

Keywords: uveal melanocytes, ovine, sheep, in vitro culture, cell isolation, spheroid, 3D culture, isolation of uveal melanocyte, 3d culture of ovine uveal melanocyte, ovine eyes uveal melanocyte, ovine uveal melanocyte, isolated uveal melanocyte, successful isolation of uveal melanocyte, available sources of uveal melanocyte, 3d growth of isolated uveal melanocyte, uveal melanocyte, measurement of melanin content, melanin content, cells from human, uvea, present in the uvea, immunohistochemistry

Abstract

Uveal melanocytes can be difficult to isolate and culture *in vitro* due to the number of cell types present in the uvea. Methodologies have been described to isolate these cells from humans, macaques and canines. This process has, however, not been described from other more readily available sources of uveal melanocytes, including the modification and establishment of protocols for 2D and 3D growth of isolated uveal melanocytes.

In this protocol, we describe a technique for extraction, isolation and both 2D and 3D culture of ovine uveal melanocytes, derived from an abattoir source. Methods to confirm the successful isolation of uveal melanocyte by PCR, immunohistochemistry and measurement of melanin content are described.

Globe Collection

1 Obtain sheep eyeballs from a local abattoir and keep on ice for transport to the laboratory. Dissection of eyeballs should be done between 2-3 hours post-mortem.

2 Before dissection, trim residual tissue and then spray eyeball with 70% ethanol to prevent fungal infection in cell culture (**Figure 1**).

Figure 1: The various sections of the dissected eye.

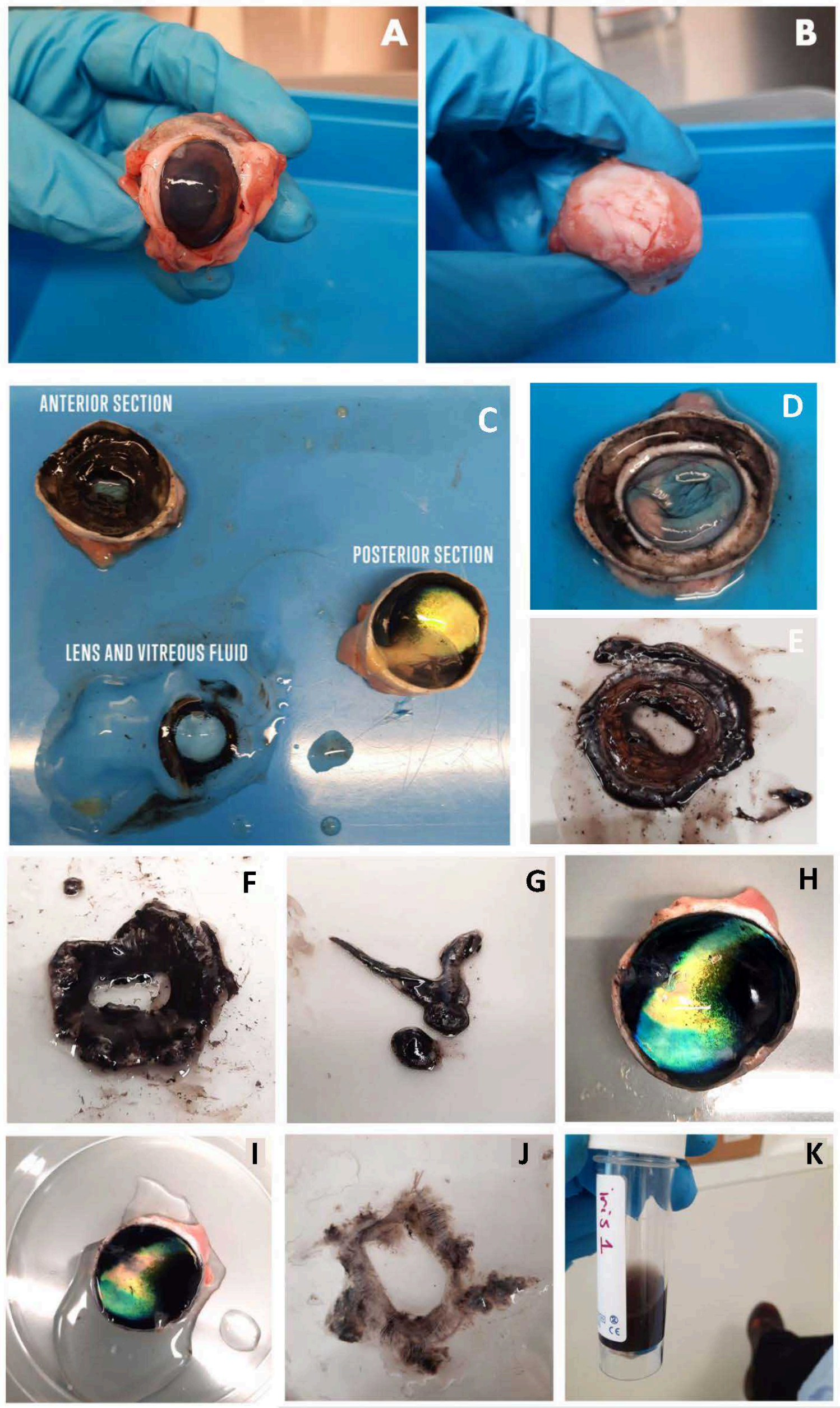

Figure 1: Front (A) and back (B) of a sheep eye before dissection. (C)The anterior and posterior sections and removed lens and vitreous fluid from the eye. (D) The remainder of the anterior section once the iris and ciliary body have been removed. (E) The iris anterior side up. (F) The iris posterior side up. This still has the ciliary body attached. (G) The ciliary body was removed and put into a separate petri dish. (H) The posterior section of the eyeball after the retina has been removed. (I) The posterior cup filled with trypsin. (J) The iris was incubated with 0.25% trypsin for 1 hour and the iris stroma was separated (K) The iris melanocyte cells captured in the trypsin, ready for the next step of the protocol.

Uveal melanocyte isolation

3 Make an initial incision through the sclera with a scalpel then cut the globe circumferentially into the anterior and posterior sections using scissors (**Figure 1**).

4 Place the anterior and posterior sections in culture dishes and remove the vitreous fluid and lens (**Figure 1**).

5 ***Iris and ciliary body sections***

Using tissue dressing forceps, remove the iris and ciliary body together from the anterior section and place in a fresh culture dish (**Figure 1**). The ciliary body can be distinguished from the iris by the difference in colour between the tissues when viewing the anterior side of the removed tissues; the iris is visibly brown in contrast to the black surrounding tissues, the ciliary body.

6 ***Choroidal section***

Using dissecting forceps, remove the retina from the posterior section of the globe, which reveals the RPE layer, including the tapetum lucidum **(Figure 1)**.

7 To isolate the RPE cells, fill the posterior section with 0.125% trypsin-EDTA solution and incubate for 1 hour at 37°C **(Figure 1).**

8 Remove the RPE from the posterior cup by agitation, with forceps, until it disperses into solution **(Figure 1)** and prepare for culture**. See section below 'Isolation and culture of RPE cells'.**

9 Remove the choroid from the sclera and discard the sclera.

10 The iris stroma, ciliary body, and choroid (collectively, the 'uveal stroma') requires successive incubations and cell release, as follows:

10.1 Incubate the uveal stroma for 18 hours at 4°C with 3 mL 0.25% trypsin. Then add 6 mL F-12 Ham's medium (Fisher Scientific) with 10% Foetal Bovine Serum (FBS; Fisher Scientific) to inactivate the trypsin. Collect the released cells and media, centrifuge at 300g for 5 minutes, then resuspend in 6 mL FIC medium (**Table 1** below).

10.2 Wash the uveal stroma with HBSS and incubate with 5 mL collagenase (1.27 mg/mL; Gibco™) in F12 medium with 10% FBS at 37°C for 1 hour. Collect the released cell pellet

(300g for 5 minutes) and resuspend in 6 mL complete medium into three wells of a 6-well plate.

10.3 Perform another collagenase incubation, as described in previous step, to maximise the yield. Seed the cells separately into three wells of a 6-well plate to minimise contamination potential.

In all three steps above, geneticin should be added to the culture media (final concentration 100 µg/mL) for 7 days to inhibit fibroblasts; thereafter, culture cells in medium without geneticin.

11 Change media approximately twice a week and cryopreserve cells in media containing 10% DMSO.

Investigate optimal media for culturing uveal melanocytes

12 Background information: Early studies showed that the addition of 12-O-Tetradecanoylphorbol-13-acetate (TPA) (also known as PMA; phorbol 12-myristate 13-acetate) and cholera toxin to culture medium aided growth of skin melanocytes. The effect of PMA and cholera toxin on uveal melanocytes showed that cell numbers increased, whilst the growth of contaminating cells decreased. Human uveal melanocytes are often grown in FIC medium, another outcome of the Hu et al 1993 protocol, which tested various combinations of supplements added to F12 medium.

In this protocol, in addition to FIC medium, we test complete smooth muscle growth medium (SMGM), which was selected as it consistently improved growth rates across commercially available human uveal melanoma cell lines in the Cancer Cell Line Factory (CCLF)/DepMap project.

Epidermal growth factor (EGF) and insulin have previously been added to culture media for the growth of skin melanocytes and EGF was included in the CCLF media optimisations. Therefore in this protocol we investigated the addition of 10 µg/mL human insulin (I2643, Sigma) and 10 ng/mL EGF Recombinant Human Protein (Gibco™; Fisher) to the SMGM media.

13

	A	B	C	D
	**Reagent**	**Manufacturer/ Supplier**	**Cat No.**	**Final Concentration**
	**FIC media**			

	A	B	C	D
	Gibco Ham's F-12 Nutrient Mix	Fisher/Gibco	F1211765054	n/a
	Gibco FBS	Fisher/Gibco	11573397	10%
	Cholera Toxin	Scientific Laboratory Supplies Ltd	C8052-.5MG	10ng/mL
	MP Biomedicals Gentamicin Sulfate	Fisher	11480435	50µg/mL
	Alfa Aesar IBMX	Fisher	15466479	0.1mM
	Human FGF-basic Recombinant Protein	Fisher	PHG0021	20ng/mL
	Glutamine	Already in the F12 media	n/a	n/a
	*Gibco Geneticin Selective Antibiotic	Fisher/Gibco	11558616	100µg/mL
	**Smooth Muscle Growth Media**			
	Smooth Muscle Cell Growth Medium	Cell Applications, Inc / Sigma	311-500	n/a
	Gibco FBS	Fisher/Gibco	11573397	10%
	L-Glutamine	Gibco	25030081	2mM
	Human insulin (*optional additional tested additive*)	Sigma	I2643	10µg/mL

	A	B	C	D
	EGF Recombinant Human Protein (*optional additional tested additive*)	Gibco	PHG0311	10ng/mL

Table 1: Components of FIC media and smooth muscle growth media (SMGM) tested for growth of ovine melanocytes.
*geneticin was only added to media for first 3-7days of culture. IBMX, 3-Isobutyl-1-methylxanthine; FBS, Fetal Bovine Serum; FGF, fibroblast growth factor; EGF, epithelial growth factor

Measurement of melanin concentration of uveal melanocytes

14 To spectrophotometrically quantify total melanin content per cell, synthetic melanin can be used as a standard.

15 Seed uveal melanocytes into 12-well plates.

16 Three, five and twelve days after seeding, trypsin cells and count using a haemocytometer.

17 Pellet the cells (centrifuge 500g for 5 minutes) and lyse with 1% Triton-X100 in PBS for 30 minutes at 4°C, then pellet again (500g at 4°C for 30 minutes).

18 Remove the soluble fraction, and measure protein content using the Micro Total Protein Kit (Sigma, UK).

19 Solubilise the insoluble fraction containing the melanin by adding 400 µL 1N NaOH and incubate for 1 hour at 85°C.

20 Immediately read this fraction at λ = 405nm (Varioskan™ LUX multimode microplate reader (ThermoFisher, UK)) and measure melanin content against a standard curve for synthetic melanin (M8631, Sigma, UK) at a range of 0.012 – 100 µg/mL.

Confirmation of isolation of uveal melanocytes by immunohistochemistry

21 Plate uveal melanocytes or RPE primary cells isolated from sheep eyes or A2058 cutaneous melanoma cell lines on glass coverslips placed in 6-well plates.

22 Grow cells to 50-70% confluency, wash twice in PBS and fix in absolute ethanol plus 5% glacial acetic acid at -20°C for 10 minutes.

23 Wash the coverslips three times with PBS.

24 Block with 1% BSA in PBS for 1 hour at room temperature.

25 Incubate the coverslips overnight at 4°C with the primary antibody (**Table 2**). No appropriate sheep specific antibodies were available, so antibody targets were selected after ensuring the homology of sheep to human proteins was ≥95%.

	A	B	C	D	E	F
	**Antibody**	**Host; Specificity**	**Reported Cross Reactivity**	**Isotype, Clone**	**Dilutions Tested**	**Catalogue Number**
	S100B	Rabbit, anti-human	Mouse, Rat	IgG, EP1576Y	1:200 and 1:600	Ab52642
	SOX10	Rabbit, anti-human	Mouse, Rat	IgG, EPR4007-104	1:100 and 1:300	Ab180862
	HRP conjugated anti-rabbit IgG	Goat, Rabbit	-	IgG (polyclonal)	1:500	Ab97051

Table 2: Antibodies used for immunohistochemistry

26 Dilute primary antibodies in 1% BSA in PBS as per **Table 2**.

27 For the negative control, incubate coverslips with 1% BSA in PBS alone, then wash three times with Tris-buffered saline (TBS) 0.025% Triton-X100.

28 Then incubate coverslips with 0.3% H_2O_2 in TBS for 15 minutes to block endogenous peroxidase activity.

29 Dilute HRP conjugated anti-rabbit IgG (Abcam, ab97051) 1:500 in TBS and add to slides for 1 hour at room temperature.

30 Wash coverslips three times with PBS and incubate with 3,3'-Diaminobenzidine (DAB) substrate (Abcam, ab64238) for 10 minutes.

31 Wash coverslips with tap water, then counterstain with Mayer's haematoxylin (Abcam, ab220365) for 30 seconds and wash again with tap water.

32 Mount coverslips onto microscope slides and analysed using a light microscope (Motic, AE31E; image software, Motic images plus 2.0).

Confirmation of the isolation of uveal melanocytes by measurement of melanin concentration

33 To further confirm isolation of uveal melanocytes, the active expression of the genes encoding tyrosinase (*TYR*), pre3elanosome protein (*PMEL*) and melan-A (*MLANA*) can be assessed by the PCR of mRNA.

34 Extract the mRNA of melanocyte cultures using the RNeasy Plus Kit (Qiagen, UK).

35 Convert 1mg of mRNA to cDNA using the GoScript™ Reverse Transcriptase kit with a mixture of random hexamers and oligo d(T) primers (Promega, UK), following the manufacturers protocol.

36 Perform the polymerase chain reaction (PCR) using GoTaq® qPCR kit (Promega, UK) following the manufacturers protocol.

37 The primers for Tyrosinase (*TYR*), premelanosome protein (*PMEL*) and melan-A (*MLANA*) are detailed in **Table 3**. These primers were designed using Primer3 (**https://primer3.ut.ee/**) specific to the ovine mRNA sequences and spanning an intron to ensure specificity to the mRNA sequence.

	A	B	C	D
	**Gene Target**	**Forward Primer**	**Reverse Primer**	**Spanning intron size**
	Tyrosinase (TYR)	GCACAACCGGGAATCCTACA	GGCTAGTAAGTCCTCCCAGC	13,562bp
	Premelanosome protein (PMEL)	GGTTACACGGGGAACAACTC	GGTGAGGGAAAAGGAGCCAT	927bp
	Melan-A (MLANA)	GGCATCCTGACAGTGATTCT	TGTTGACCAAGCCACTCATG	6,784

Table 3: Primers used for PCR

38 Carry out PCR on a StepOne system (Applied Biosystems, UK) and inspect melt curves to ensure primer specificity and single product production.

3D cell culture of uveal melanocyte spheroids

39 The creation of 3D spheroids of uveal melanocytes can be investigated using various methods including the hanging drop method, agar-coated plates, ultra-low attachment plates and round-bottom plates.

40 **Hanging drop method**

Pipette 10 or 20 µL droplets of a 0.25-2 × 10^6 cell/ml suspension onto the lid of a culture plate.

40.1 Invert the lid and place back onto the plate so that droplets are suspended.

40.2 Fill the wells with PBS to prevent the droplets from drying.

40.3 After 24 or 48 hours, transfer the spheroids from the droplet to either ultra-low attachment plates or agar-coated wells, using a P20 with the tip cut.

41 ***Round-bottom plates***
Seed cells at 6000, 10,000 and 20,000 cells per well in BIOFLOAT 96-well round-bottom plates (faCellitate, Germany).

42 ***Methyl cellulose***
Methyl cellulose can be added to culture media to test whether it promotes spheroid formation. To test this, prepare a stock of 100 mg/mL methyl cellulose by heating 50 mL of ultrapure water (Invitrogen) to 80°C and adding 10g of methyl cellulose (M7027, Sigma).

42.1 Add cold ultrapure water to the solution to a final volume of 100 mL and incubate on a shaker at 4°C overnight.

42.2 Use methyl cellulose at a final concentration of 0.2, 1 and 2% in culture medium (based on previous studies, including on renal cancer carcinoma cell lines), and pass through a 0.2 µm sterile filter before use.

Isolation and culture of RPE cells

43 To isolate RPE cells, remove the trypsin from the incubation in the posterior eye cup, as described in step 7.

44 Add culture media with 10% FBS to stop the trypsin reaction and pellet cells (centrifuge at 300g for 5 minutes).

45 Then resuspend the pellet in 6 mL DMEM plus 10% FBS.

46 Seed the RPE cells across three wells of a 6-well plate.

47 Once the isolated cells reach ~70-80% confluency, they can be cryopreserved in DMEM plus 10% FBS containing 10% DMSO for future experiments.

Protocol references

1. Brenner M, Hearing VJ. The protective role of melanin against UV damage in human skin. Photochem Photobiol. 2008;84(3):539-49.
2. Boulton ME. Studying melanin and lipofuscin in RPE cell culture models. Exp Eye Res. 2014;126:61-7.
3. Boulton M, Dayhaw-Barker P. The role of the retinal pigment epithelium: topographical variation and ageing changes. Eye (Lond). 2001;15(Pt 3):384-9.
4. Hu DN, McCormick SA, Orlow SJ, Rosemblat S, Lin AY, Wo K. Melanogenesis by human uveal melanocytes in vitro. Invest Ophthalmol Vis Sci. 1995;36(5):931-8.
5. Hu F, Teramura DJ, Mah K. Normal uveal melanocytes in culture. Pigment Cell Res. 1987;1(2):94-103.
6. Hu DN, McCormick SA, Ritch R, Pelton-Henrion K. Studies of human uveal melanocytes in vitro: isolation, purification and cultivation of human uveal melanocytes. Invest Ophthalmol Vis Sci. 1993;34(7):2210-9.
7. Hu D-N, Mccormick SA, Seedor JA, Ritterband DC, Shah MK. Isolation, purification and cultivation of conjunctival melanocytes. Experimental Eye Research. 2007;84(4):655-62.
8. Mouriaux F, Casagrande F, Pillaire MJ, Manenti S, Malecaze F, Darbon JM. Differential expression of G1 cyclins and cyclin-dependent kinase inhibitors in normal and transformed melanocytes. Invest Ophthalmol Vis Sci. 1998;39(6):876-84.
9. Li L, Hu D-N, Zhao H, Mccormick SA, Nordlund JJ, Boissy RE. Uveal Melanocytes Do Not Respond To or Express Receptors for α-Melanocyte-Stimulating Hormone. Investigative Opthalmology & Visual Science. 2006;47(10):4507.
10. Calipel A, Abonnet V, Nicole O, Mascarelli F, Coupland SE, Damato B, et al. Status of RASSF1A in Uveal Melanocytes and Melanoma Cells. Molecular Cancer Research. 2011;9(9):1187-98.
11. Weidmann C, Pomerleau J, Trudel-Vandal L, Landreville S. Differential responses of choroidal melanocytes and uveal melanoma cells to low oxygen conditions. Mol Vis. 2017;23:103-15.
12. Sitiwin E, Madigan MC, Gratton E, Cherepanoff S, Conway RM, Whan R, et al. Shedding light on melanins within in situ human eye melanocytes using 2-photon microscopy profiling techniques. Scientific Reports. 2019;9(1).
13. Dawson-Baglien EM, Winkler PA, Bruewer AR, Petersen-Jones SM, Bartoe JT. Isolation and cultivation of canine uveal melanocytes. Veterinary Ophthalmology. 2015;18(4):285-90.
14. Eisinger M, Marko O. Selective proliferation of normal human melanocytes in vitro in the presence of phorbol ester and cholera toxin. Proceedings of the National Academy of Sciences of the United States of America. 1982;79(6):2018-22.
15. An J, Wan H, Zhou X, Hu D-N, Wang L, Hao L, et al. A Comparative Transcriptomic Analysis of Uveal Melanoma and Normal Uveal Melanocyte. PLoS ONE. 2011;6(1):e16516.
16. Walker TM. Expression of Angiogenic Factors Cyr61 and Tissue Factor in Uveal Melanoma. Archives of Ophthalmology. 2002;120(12):1719.
17. Bergeron MA, Champagne S, Gaudreault M, Deschambeault A, Landreville S. Repression of genes involved in melanocyte differentiation in uveal melanoma. Mol Vis. 2012;18:1813-22.
18. Zuidervaart W, Van Der Velden PA, Hurks MH, Van Nieuwpoort FA, Out-Luiting CJJ, Singh AD, et al. Gene expression profiling identifies tumour markers potentially playing a role in uveal melanoma development. British

Journal of Cancer. 2003;89(10):1914-9.
19. Ghandi M, Huang FW, Jane-Valbuena J, Kryukov GV, Lo CC, McDonald ER, 3rd, et al. Next-generation characterization of the Cancer Cell Line Encyclopedia. Nature. 2019;569(7757):503-8.
20. Szabad G, Kormos B, Pivarcsi A, Széll M, Kis K, Kenderessy Szabó A, et al. Human adult epidermal melanocytes cultured without chemical mitogens express the EGF receptor and respond to EGF. Archives of Dermatological Research. 2007;299(4):191-200.
21. Oancea E, Vriens J, Brauchi S, Jun J, Splawski I, Clapham DE. TRPM1 forms ion channels associated with melanin content in melanocytes. Sci Signal. 2009;2(70):ra21.
22. Matak D, Brodaczewska KK, Lipiec M, Szymanski Ł, Szczylik C, Czarnecka AM. Colony, hanging drop, and methylcellulose three dimensional hypoxic growth optimization of renal cell carcinoma cell lines. Cytotechnology. 2017;69(4):565-78.
23. Leung BM, Lesher-Perez SC, Matsuoka T, Moraes C, Takayama S. Media additives to promote spheroid circularity and compactness in hanging drop platform. Biomater Sci. 2015;3(2):336-44.
24. Maritan SM, Lian EY, Mulligan LM. An Efficient and Flexible Cell Aggregation Method for 3D Spheroid Production. Journal of Visualized Experiments. 2017(121).
