# Supplementary material for: Using ovine eyes as a readily available source for the isolation and 2D or 3D cultivation of uveal melanocytes
Source: PLoS One. 2026 Sep 21;21(9):e0358056. doi: 10.1371/journal.pone.0358056 (PMC13592627; doi:10.1371/journal.pone.0358056)
Supplement: Supplementary file 7 [file pone.0358056.s006.docx]

Supplementary Table 1: Media used in published studies for the culture of uveal melanocytes from primary tissue

| **Culture medium** | **Cell type** | **Aim of work** | **Confirmation of cell type** | **Citation** |
| --- | --- | --- | --- | --- |
| MEM plus 20% FCS | Primary uveal melanocytes from fetal eyes | Examination of melanoma karotypes | Visually by microscope | (Giovanella et al. 1976) |
| Eagle’s MEM supplemented with 5 or 10% FBS. Sometimes with addition of TPA and cholera toxin to help remove contaminating cells. A feeder cell layer plus Humidified CO^2^ chamber at 37°C | Macaque iris and choroidal melanocytes | Culture of uveal melanocytes | Visual by microscope | (Hu, Teramura, and Mah 1987) |
| Comparison of three culture media   - F12 medium: F-12 media supplemented with 10% FBS, gentamicin (50 µg/mL) and glutamine (2 mM) - TIC medium: F12 medium as above with addition of 100nM 12-0-tetradeca-noyl phorbol 13-acetate (TPA), 0.1 mM IBMX and 10ng/mL cholera toxin - FIC medium: TIC medium as above, but instead of TPA, 20 ng/mL hFGF is added | Primary uveal melanocytes from adult human donors | Protocol for isolation and purification of human uveal melanocytes | Visually/Immunocytochemical staining of S-100, vimentin and cytokeratin | (Hu et al. 1993) |
| MCDB153 culture medium18 supplemented with bovine insulin (10 µg/mL), human transferrin (10 µg/mL), hydrocortisone (2.8 µg/mL), glutamine (2 mM), nystatin (10 IU/mL), vitamin E (1 ng/mL), phorbol myristate acetate (PMA; 10 nM), cholera toxin (100 ng/mL), bovine hypothalamic extract (100 µg/mL), chelexed fetal calf serum (2%). Medium is based on skin melanocyte culture (Pittelkow and Shipley 1989) | Human choroidal melanocytes | Effect of MSH on uveal melanocytes and uveal melanoma cells | Visually by microscope/ DOPA-staining | (Goodall et al. 1994) |
| Choroidal melanocytes were cultured in Ham's F12 medium (pH 7.3), supplemented with 100 IU/mL penicillin, 100 µg/mL streptomycin, 2.5 µg/mL amphotericin B, 2 mM L-glutamine, 0.1 mM IBMX, 10 ng/mL cholera toxin, 5 ng/mL basic fibroblast growth factor-2, and 10% fetal calf serum. To ensure elimination of these cells, 100 µg/mL neomycin was also added during the first days of culture. | Human choroidal melanocytes | Investigation of levels of cell cycle regulators in normal and transformed melanocytes | HMB-45 and S100 staining | (Mouriaux et al. 1998) |
| **Culture medium** | **Cell type** | **Aim of work** | **Confirmation of cell type** | **Citation** |
| (1) MCDB153 medium supplemented with bovine insulin (10 µg/ml), human transferrin (10 µg/mL), hydrocortisone (2.8 µg/mL), glutamine (2 mM), vitamin E (1 µg/mL), phorbol myristate acetate (10 nM), cholera toxin (100 ng/mL), bovine pituitary extract (50 ng/mL) and chelexed FCS (2%) (19). The medium contained L-tyrosine at 0.003 g/L. Slight variation on medium used by (Goodall et al. 1994)  (2) Green’s medium (media used in this study for keratinocyte culture); DMEM and Ham’s F12 medium in a 3:1 ratio supplemented with 10% FCS, 2.5 µg/mL hydrocortisone, 10 ng/mL EGF, 10−10 mol/L cholera toxin, 1.8× 10−14 mol/L adenine, 5 µg/mL insulin, 5 µg/mL transferrin, 2×10−3 mol/L glutamine, 2×10−7 mol/L triiodo-L-thyronine, 0.625 µg/mL amphotericin B, 100 U/ml penicillin and 100 µg/mL streptomycin) | Human choroidal/iridial melanocytes | Hormonal/molecular regulation of pigmentation | Morphology/Immunocytochemical detection of TRP1 and MC-1R | (Smith-Thomas et al. 2001) |
| FIC medium (F-12 Ham's medium supplemented with 10% fetal calf serum (FCS), 2.5 mg/mL fungizone/amphotericin B, 2 mmol/L L-glutamine, 10 ng/mL cholera toxin, 0.1 mmol/L IBMX, and 5 ng/mL FGF2. | Human choroidal/iridial melanocytes | Effect of α-MSH on uveal melanocytes | Not specified | (Li et al. 2006) |
| FIC medium (F-12 Ham's medium supplemented with 10% fetal calf serum (FCS), 2.5 mg/mL fungizone/amphotericin B, 2 mmol/L L-glutamine, 10 ng/mL cholera toxin, 0.1 mmol/L IBMX, and 5 ng/mL FGF2. | Human uveal melanocytes | RASSF1a expression in uveal melanoma vs uveal melanocytes | Not specified | (Calipel et al. 2011) |
| FNC Coating Mix and Ham’s F12 medium supplemented with 10% FBS, 10 ng/mL cholera toxin, 100 nM phorbol 12-myristate 13-acetate, 100 µg/mL geneticin, and 50µg/mL gentamicin. | Human Choroidal melanocytes | Comparison between 21% and 3% oxygen conditions on uveal melanocytes | Microscopic inspection and Melan-A staining | (Weidmann et al. 2017) |
| Melanocyte growth media (not commercial): 10% FBS in Ham’s F12 with 2 mM L-glutamine, 0.1 mM IBMX, 100 nM Phorbol 12-myristate 13-acetate (PMA), 10 ng/mL cholera toxin | Human choroidal melanocytes | Method development for *in situ* profiling of melanin in choroidal melanocytes | 2-photon excitation fluorescence of to identify melanin | (Sitiwin et al. 2019) |
| Melanocyte growth medium M2 medium (PromoCell) | Human choroidal melanocytes | Protein signatures of extracellular vesicles from primary normal uveal melanocytes and uveal melanoma cell lines | Morphology and pigmentation, immunofluorescent staining | (Tsering et al. 2020) |

FBS, fetal bovine serum; IBMX, isobutylmethylxanthine; hFGF, human fibroblast growth factor; MSH, melanocyte stimulating hormone; TPA, Tetradecanoylphorbol-13-acetate; DMEM, Dulbecco's Modified Eagle Medium; RPE cells, retinal pigment epithelial cells, EGF, epidermal growth factor; MEM, Modified Eagle Medium.

**References**

Calipel, Armelle, Véronique Abonnet, Olivier Nicole, Frédéric Mascarelli, Sarah E. Coupland, Bertil Damato, and Frédéric Mouriaux. 2011. 'Status of RASSF1A in Uveal Melanocytes and Melanoma Cells', *Molecular Cancer Research*, 9: 1187-98.

Giovanella, B. C., J. S. Stehlin, C. Santamaria, S. O. Yim, A. C. Morgan, L. J. Williams, A. Leibovitz, P. J. Fialkow, and D. M. Mumford. 1976. 'Human neoplastic and normal cells in tissue culture. I. Cell lines derived from malignant melanomas and normal melanocytes', *J Natl Cancer Inst*, 56: 1131-42.

Goodall, T., J. A. Buffey, I. G. Rennie, M. Benson, M. A. Parsons, M. K. Faulkner, and S. MacNeil. 1994. 'Effect of melanocyte stimulating hormone on human cultured choroidal melanocytes, uveal melanoma cells, and retinal epithelial cells', *Invest Ophthalmol Vis Sci*, 35: 826-37.

Hu, D. N., S. A. McCormick, R. Ritch, and K. Pelton-Henrion. 1993. 'Studies of human uveal melanocytes in vitro: isolation, purification and cultivation of human uveal melanocytes', *Invest Ophthalmol Vis Sci*, 34: 2210-9.

Hu, F., D. J. Teramura, and K. Mah. 1987. 'Normal uveal melanocytes in culture', *Pigment Cell Res*, 1: 94-103.

Li, Li, Dan-Ning Hu, Huiquan Zhao, Steven A. Mccormick, James J. Nordlund, and Raymond E. Boissy. 2006. 'Uveal Melanocytes Do Not Respond To or Express Receptors for α-Melanocyte-Stimulating Hormone', *Investigative Opthalmology & Visual Science*, 47: 4507.

Mouriaux, F., F. Casagrande, M. J. Pillaire, S. Manenti, F. Malecaze, and J. M. Darbon. 1998. 'Differential expression of G1 cyclins and cyclin-dependent kinase inhibitors in normal and transformed melanocytes', *Invest Ophthalmol Vis Sci*, 39: 876-84.

Pittelkow, M. R., and G. D. Shipley. 1989. 'Serum-free culture of normal human melanocytes: growth kinetics and growth factor requirements', *J Cell Physiol*, 140: 565-76.

Sitiwin, Ephrem, Michele C. Madigan, Enrico Gratton, Svetlana Cherepanoff, Robert Max Conway, Renee Whan, and Alexander Macmillan. 2019. 'Shedding light on melanins within in situ human eye melanocytes using 2-photon microscopy profiling techniques', *Scientific Reports*, 9.

Smith-Thomas, L. C., M. Moustafa, R. A. Dawson, M. Wagner, C. Balafa, J. W. Haycock, A. H. Krauss, D. F. Woodward, and S. MacNeil. 2001. 'Cellular and hormonal regulation of pigmentation in human ocular melanocytes', *Pigment Cell Res*, 14: 298-309.

Tsering, T., A. Laskaris, M. Abdouh, P. Bustamante, S. Parent, E. Jin, S. T. Ferrier, G. Arena, and J. V. Burnier. 2020. 'Uveal Melanoma-Derived Extracellular Vesicles Display Transforming Potential and Carry Protein Cargo Involved in Metastatic Niche Preparation', *Cancers (Basel)*, 12.

Weidmann, C., J. Pomerleau, L. Trudel-Vandal, and S. Landreville. 2017. 'Differential responses of choroidal melanocytes and uveal melanoma cells to low oxygen conditions', *Mol Vis*, 23: 103-15.
