# Supplementary material for: Using ovine eyes as a readily available source for the isolation and 2D or 3D cultivation of uveal melanocytes
Source: PLoS One. 2026 Sep 21;21(9):e0358056. doi: 10.1371/journal.pone.0358056 (PMC13592627; doi:10.1371/journal.pone.0358056)
Supplement: Supplementary file 8 [file pone.0358056.s007.docx]

Supplementary Table 2:

Components of FIC media and smooth muscle growth media (SMGM) tested for growth of ovine melanocytes.

| **Reagent** | **Manufacturer/**  **Supplier** | **Cat No.** | **Final Concentration** |
| --- | --- | --- | --- |
| **FIC media** |  |  |  |
| Gibco™ Ham's F-12 Nutrient Mix | Fisher/Gibco | F1211765054 | n/a |
| Gibco™ FBS | Fisher/Gibco | 11573397 | 10% |
| Cholera Toxin | Scientific Laboratory Supplies Ltd | C8052-.5MG | 10ng/mL |
| MP Biomedicals Gentamicin Sulfate | Fisher | 11480435 | 50µg/mL |
| Alfa Aesar™  IBMX | Fisher | 15466479 | 0.1mM |
| Human FGF-basic Recombinant Protein | Fisher | PHG0021 | 20ng/mL |
| Glutamine | Already in the F12 media | n/a | n/a |
| *Gibco™ Geneticin Selective Antibiotic | Fisher/Gibco | 11558616 | 100µg/mL |
|  |  |  |  |
| **Smooth Muscle Growth Media** |  |  |  |
| Smooth Muscle Cell Growth Medium | Cell Applications, Inc  / Sigma | 311-500 | n/a |
| Gibco™ FBS | Fisher/Gibco | 11573397 | 10% |
| L-Glutamine | Gibco | 25030081 | 2mM |
| Human insulin  (*optional additional* *tested additive*) | Sigma | I2643 | 10μg/mL |
| EGF Recombinant Human Protein (*optional additional* *tested additive*) | Gibco | PHG0311 | 10ng/mL |

*geneticin was only added to media for first 3-7days of culture. IBMX, 3-Isobutyl-1-methylxanthine; FBS, Fetal Bovine Serum; FGF, fibroblast growth factor; EGF, epithelial growth factor
