# Supplementary material for: Using ovine eyes as a readily available source for the isolation and 2D or 3D cultivation of uveal melanocytes
Source: PLoS One. 2026 Sep 21;21(9):e0358056. doi: 10.1371/journal.pone.0358056 (PMC13592627; doi:10.1371/journal.pone.0358056)
Supplement: Supplementary file 9 [file pone.0358056.s008.docx]

**Supplementary Materials and Methods**

*Isolation and culture of RPE cells*

RPE cells were obtained by removing the trypsin from the incubation in the posterior eye cup, as described in the ‘Materials and Methods’. Culture media with 10% FBS was added to stop the trypsin reaction and cells pelleted (300g for 5 minutes) then resuspended in 6 mL DMEM plus 10% FBS. RPE cells were seeded across three wells of a 6-well plate. Once the isolated cells reached ~70-80% confluency, they were cryopreserved in DMEM plus 10% FBS containing 10% DMSO.

*Immunohistochemistry*

Uveal melanocytes or RPE primary cells isolated from sheep eyes or A2058 cutaneous melanoma cell lines were plated on glass coverslips placed in 6-well plates. The cells were grown to 50-70% confluency, washed twice in PBS and fixed in absolute ethanol plus 5% glacial acetic acid at -20°C for 10 minutes. The coverslips were then washed three times with PBS and blocked with 1% BSA in PBS for 1 hour at room temperature. The coverslips were incubated overnight at 4°C with the primary antibody (Supplementary Materials Table 1). No appropriate sheep specific antibodies were available, so antibody targets were selected after ensuring the homology of sheep to human proteins was ≥95%. All primary antibodies were diluted in 1% BSA in PBS, as per Supplementary Table 1. For the negative control, coverslips were incubated with 1% BSA in PBS alone, then washed three times with Tris-buffered saline (TBS) 0.025% Triton-X100. Coverslips were incubated with 0.3% H_2_O_2_ in TBS for 15 minutes to block endogenous peroxidase activity. HRP conjugated anti-rabbit IgG (Abcam, ab97051), was diluted 1:500 in TBS and added to slides for 1 hour at room temperature. Coverslips were washed three times with PBS and incubated with 3,3'-Diaminobenzidine (DAB) substrate (Abcam, ab64238) for 10 minutes. Coverslips were washed with tap water, counterstained with Mayer’s haematoxylin (Abcam, ab220365) for 30 seconds and washed again with tap water. Coverslips were mounted onto microscope slides and analysed using a light microscope (Motic, AE31E; image software, Motic images plus 2.0).

Supplementary Materials Table 1: Antibodies used for immunohistochemistry

| **Antibody** | **Host; Specificity** | **Reported Cross Reactivity** | **Isotype, Clone** | **Dilutions Tested** | **Catalogue Number** |
| --- | --- | --- | --- | --- | --- |
| S100B | Rabbit, anti-human | Mouse, Rat | IgG, EP1576Y | 1:200 and 1:600 | Ab52642 |
| SOX10 | Rabbit, anti-human | Mouse, Rat | IgG, EPR4007-104 | 1:100 and 1:300 | Ab180862 |
| HRP conjugated anti-rabbit IgG | Goat, Rabbit | - | IgG (polyclonal) | 1:500 | Ab97051 |

*Polymerase Chain Reaction*

The mRNA of melanocyte cultures was extracted using the RNeasy Plus Kit (Qiagen, UK) and 1μg of mRNA was converted to cDNA using the GoScript™ Reverse Transcriptase kit with a mixture of random hexamers and oligo d(T) primers (Promega, UK), following the manufacturers protocol. The polymerase chain reaction (PCR) was performed using GoTaq® qPCR kit (Promega, UK) following the manufacturers protocol. The primers for Tyrosinase (*TYR*), premelanosome protein (*PMEL*) and melan-A (*MLANA*) are detailed in Supplementary Materials Table 2. These primers were designed using Primer3 (<https://primer3.ut.ee/>) specific to the ovine mRNA sequences and spanning an intron to ensure specificity to the mRNA sequence. PCR was carried out on a StepOne system (Applied Biosystems, UK) and melt curves were inspected to ensure primer specificity and single product production.

Supplementary Materials Table 2: Primers used for PCR

| **Gene Target** | **Forward Primer** | **Reverse Primer** | **Spanning intron size** |
| --- | --- | --- | --- |
| Tyrosinase (TYR) | GCACAACCGGGAATCCTACA | GGCTAGTAAGTCCTCCCAGC | 13,562bp |
| Premelanosome protein (PMEL) | GGTTACACGGGGAACAACTC | GGTGAGGGAAAAGGAGCCAT | 927bp |
| Melan-A (MLANA) | GGCATCCTGACAGTGATTCT | TGTTGACCAAGCCACTCATG | 6,784 |

*Culture of 3D spheroids*

*Hanging drop method*

10 or 20 µL droplets of a 0.25-2 x 10^6^ cell/ml suspension were pipetted onto the lid of a culture plate. The lid was then inverted and placed back onto the plate so that droplets were suspended. The wells were filled with PBS to prevent the droplets from drying. After 24 or 48 hours, the spheroids were then transferred from the droplet to either ultra-low attachment plates or agar-coated wells, using a P20 with the tip cut.

*Round-bottom plates*

Cells were seeded at 6000, 10,000 and 20,000 cells per well in BIOFLOAT 96-well round-bottom plates (faCellitate, Germany).

*Methyl cellulose*

Methyl cellulose was added to culture media to test whether it promoted spheroid formation. A stock of 100 mg/mL methyl cellulose was prepared by heating 50 mL of ultrapure water (Invitrogen) to 80°C and adding 10g of methyl cellulose (M7027, Sigma) [1]. Cold ultrapure water was added to the solution to a final volume of 100 mL and incubated on a shaker at 4°C overnight. Methyl cellulose was used at a final concentration of 0.2, 1 and 2% in culture medium (based on previous studies, including on renal cancer carcinoma cell lines [2, 3]), and passed through a 0.2 µm sterile filter before use.

**References**

1.	Maritan, S.M., E.Y. Lian, and L.M. Mulligan, *An Efficient and Flexible Cell Aggregation Method for 3D Spheroid Production.* Journal of Visualized Experiments, 2017(121).

2.	Leung, B.M., et al., *Media additives to promote spheroid circularity and compactness in hanging drop platform.* Biomater Sci, 2015. **3**(2): p. 336-44.

3.	Matak, D., et al., *Colony, hanging drop, and methylcellulose three dimensional hypoxic growth optimization of renal cell carcinoma cell lines.* Cytotechnology, 2017. **69**(4): p. 565-578.
